# GENDER DIFFERENCES IN NURSING HOME RESIDENT QUALITY OF LIFE: WHY WOMEN DO BETTER

**DOI:** 10.1093/geroni/igz038.1866

**Published:** 2019-11-08

**Authors:** Heather Davila, Tetyana P Shippee, Weiwen Ng, Odichinma C Akosionu, Beth Virnig

**Affiliations:** 1 VA Boston Healthcare System, Boston, Massachusetts, United States; 2 University of Minnesota, Minneapolis, Minnesota, United States

## Abstract

Despite research documenting gender differences in numerous outcomes in later life, we know little about gender differences in quality of life (QoL) for older adults who receive institutional long-term care. To address this gap, this study examines the relationship between gender and nursing home (NH) residents’ QoL, including possible reasons for differences observed. We used a mixed methods design including surveys with a random sample of Minnesota NH residents using a multidimensional measure of QoL (n=8,870), resident clinical data, facility-level characteristics, and qualitative interviews with NH residents (n=64). We used mixed models and thematic analysis of resident interviews to examine possible differences in resident QoL based on gender. After controlling for individual and facility characteristics, women reported higher overall QoL than men, with men reporting significantly lower QoL in 4 of 8 QoL domains. In interviews, men noted being especially dissatisfied with facility activities, whereas women more frequently described having friends in the facility and relying on family for support. Some women viewed the NH as a place of respite and described wanting to stay long-term, even though their families asked them to return home. In contrast, men more often described the NH as necessary due to physical needs, but undesirable for long-term living. Our findings provide preliminary evidence that men and women experience QoL differently, with men reporting lower QoL in several domains. Tailoring more activities for men and finding ways to strengthen relationships for men within the facility may help reduce the gender disparities in QoL we observed.

